# Using Single Peroneal Longus Tendon Graft for Segmental Meniscus Transplantation and Revision Anterior Cruciate Ligament Combined Anterolateral Reconstruction

**DOI:** 10.3390/medicina59081497

**Published:** 2023-08-21

**Authors:** Ling Yang, Chih-Hao Chiu, Kuo-Yao Hsu, Chieh-An Chuang, Alvin Chao-Yu Chen, Yi-Sheng Chan, Cheng-Pang Yang

**Affiliations:** 1 Department of Orthopedic Surgery, Division of Sports Medicine Chang Gung Memorial Hospital and Chang Gung University College of Medicine, Linkou, Taoyuan 333, Taiwan; b101104060@tmu.edu.tw (L.Y.); joechiu0115@gmail.com (C.-H.C.); emsequoia@gmail.com (K.-Y.H.); cachuang0115@gmail.com (C.-A.C.); alvinchen@cgmh.org.tw (A.C.-Y.C.); 2 Bone and Joint Research Center, Chang Gung Memorial Hospital, Linkou, Taoyuan 333, Taiwan; yschan512@gmail.com; 3 Comprehensive Sports Medicine Center, Chang Gung Memorial Hospital, Linkou, Taoyuan 333423, Taiwan; 4 Department of Orthopedic Surgery, Chang Gung Memorial Hospital, Linkou, Keelung 204, Taiwan

**Keywords:** meniscus transplantation, meniscal allograft transplantation (MAT), autograft, anterior cruciate ligament (ACL), anterolateral ligament, autologous tendon graft

## Abstract

This case report describes a new approach to segmental meniscal reconstruction using a peroneal longus autograft in a patient with recurrent traumatic medial meniscus tear and anterior cruciate ligament reconstruction (ACLR) failure. While allograft meniscal transplantation is the preferred method for treating meniscal deficiency, its high cost and various legal regulations have limited its widespread use. Autologous tendon grafts have been proposed as a substitute for allograft meniscus transplantation, but their initial results were poor, leading to little progress in this area. However, recent animal experiments and clinical studies have demonstrated promising results in using autologous tendon grafts for meniscal transplantation, including improvements in pain and quality of life for patients. Further research is needed to evaluate the effectiveness of segmental meniscal reconstruction using autologous tendon grafts, but it could potentially lead to more accessible and cost-effective treatment options for patients with meniscal deficiency.

## 1. Introduction

The primary goal of treatment for meniscal tears is to alleviate pain, enable a return to pre-injury levels of daily living activities, and prevent early degeneration of the knee joint [1]. Currently, the main treatment options include surgical interventions such as meniscectomy and meniscus repair [1]. Over the past few decades, meniscectomy has evolved from a total to a partial procedure. It remains indicated when a tear cannot be satisfactorily sutured [2]. However, complications may include a higher risk of effusion-synovitis, premature development of osteoarthritis, worsening cartilage condition, and increased likelihood of osteophyte formation after surgery [3,4,5]. Clinical doctors also studied the relationship between pre-operative symptoms and post-operative outcomes including synovitis severity and cartilage pathology of partial meniscectomy to choose a suitable surgical method for the patient and found that meniscectomy causes worse post-operative outcomes if patients are older, obese, or with a degenerative meniscal pattern and with longer time to surgery [6]. On the other hand, while the failure of alternative surgical methods can result in total or subtotal loss of meniscal function, successful repair can preserve knee kinematics and prevent progressive osteoarthritis in a younger, athletic patient population [7]. Nevertheless, if a re-tear occurs after meniscus repair, the patient may be vulnerable to subsequent meniscectomy, which can lead to a relatively poorer prognosis [8].

Meniscus transplantation is an option for patients with prior total meniscectomy or meniscus deficiency due to other causes [9]. This procedure is also suitable for individuals with meniscal deficiency in combination with anterior cruciate ligament (ACL) deficiency, who may require concomitant ACL reconstruction [10,11]. Segmental meniscal transplantation may be indicated for symptomatic patients with a focal meniscal defect, as may be seen after a prior meniscectomy [12]. Given that meniscal allograft transplantation is not widely used due to cost and availability [13], autologous tendon grafts have been proposed as a substitute for a removed meniscus. Autografts are commonly employed in labroplasty for shoulder and hip surgeries, and in recent years, many surgeons have attempted to apply this concept to knee surgery [14]. In some case series and technical notes, tendon autografts have demonstrated the ability to form a wedge-shaped structure and alleviate pain associated with meniscus deficiency [15]. 

In the following section, we present a case of ACL re-tear and failure after two times of medial bucket-handle tear repair. The patient underwent revision ACL and anterolateral ligament reconstruction with segmental meniscus reconstruction using a single peroneal longus tendon graft.

## 2. History of Present Illness

The patient is a 26-year-old male living in Taoyuan city, Taiwan, who underwent anterior cruciate ligament (ACL) reconstruction and medial meniscus (MM) repair at a local hospital two years ago, due to a traumatic right ACL tear with a bucket-handle medial meniscus tear.

**Figure 1:**
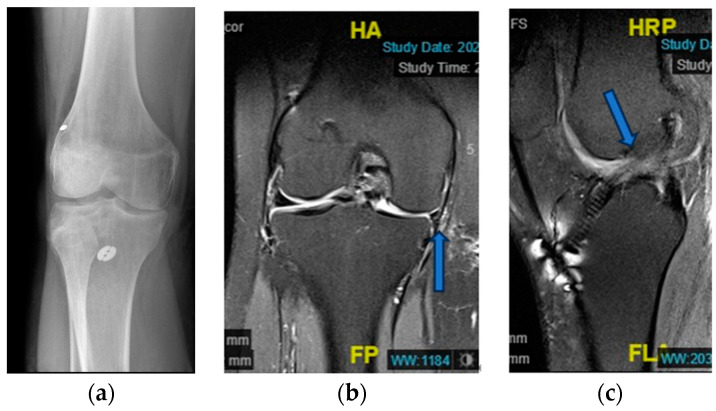
(**a**) Knee X-ray from local hospital; (**b**) MRI coronal view of knee joint, with significant medial meniscus loss (arrow); (**c**) MRI sagittal view of knee joint, ACL graft tear was noted.

However, he experienced sudden-onset medial knee pain and range of motion (ROM) limitation 1.5 years after the initial surgery. A right medial meniscus re-tear was identified, and he underwent revision MM repair at the same hospital. Six months after the second surgery, the patient was involved in a traffic accident as a motorcycle rider. He experienced severe right knee pain and was taken to the emergency room. The patient reported a locking and catching sensation in his right knee. X-ray and magnetic resonance imaging (MRI) were performed and revealed a recurrent right knee MM tear and ACL re-tear, prompting the recommendation for surgery (Figure 1).

**Figure 2:**
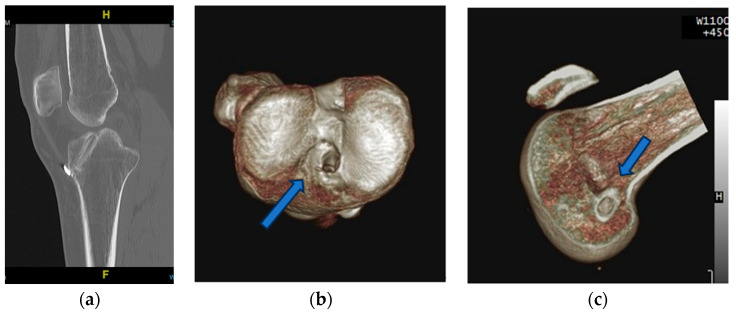
CT of right knee joint: (**a**) No tunnel widening was noted. (**b**,**c**) Previous tunnel positions were ideal. (the arrow represents the original bone tunnel).

The patient decided to transfer to our hospital for further management. At the outpatient department, physical examination revealed a grade 2 positive anterior drawer test, grade 2 positive pivot shift test, and McMurray test positive on the medial side. The right knee ROM was between 10–100 degrees. Lower extremity split scanography was performed and showed no malalignment. Revision ACL reconstruction was indicated. A 3D CT scan was then arranged, and no tunnel widening was observed. (Figure 2) According to the MRI from the previous hospital, an MM bucket-handle tear was noted. Additionally, the meniscus volume had significantly decreased. Minimal osteoarthritis changes were observed in the medial tibiofemoral compartment. After discussing with the patient, revision ACL reconstruction and meniscus transplantation were indicated. However, due to the high cost, the patient declined to use an allograft. Ultimately, we decided to use an autologous tendon graft. His demographic data is listed in Table 1. 

## 3. Surgical Procedures

**Figure 3:**
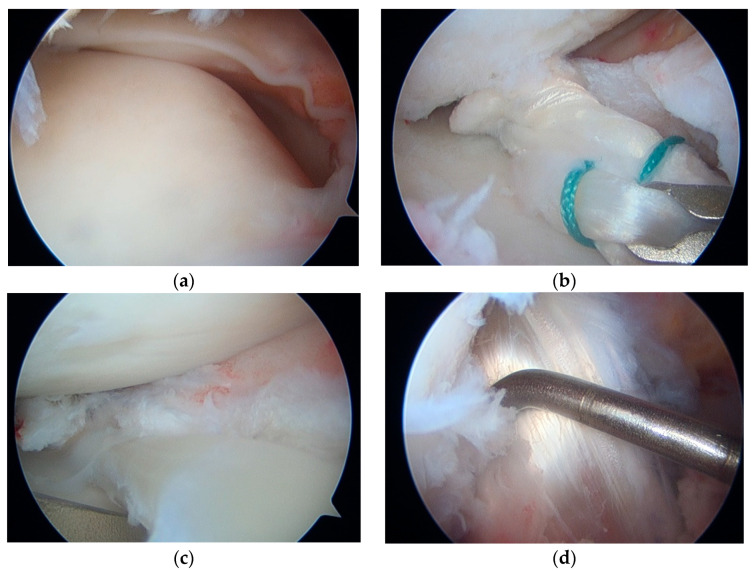
(**a**) Midbody meniscus loss was noted; (**b**) Shuttle the tendon graft to the deficient area; (**c**) Image after meniscus transplant; (**d**) Testing graft tension after ACL reconstruction.

**Figure 4:**
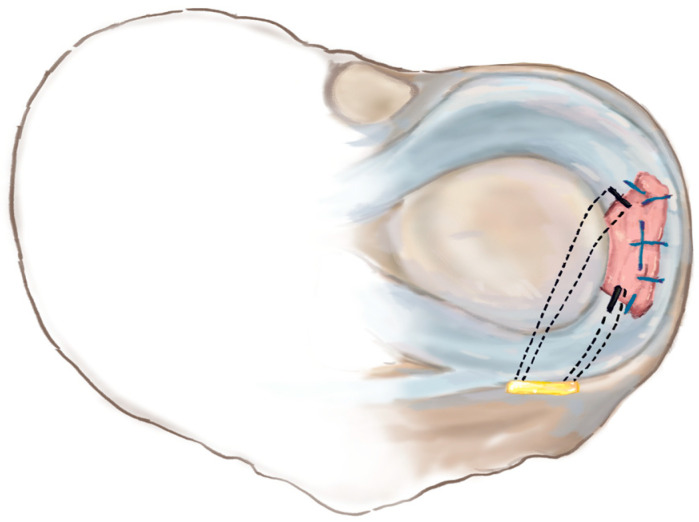
The schematic diagram of the surgical technique.

The patient was positioned supine with a leg holder. The peroneal longus autograft was harvested at full length from the insertion site using a tendon stripper. Standard anteromedial and anterolateral portals were created. Following a standard diagnostic procedure, ACL graft failure and MM mid-body loss were confirmed, while the anterior horn and posterior horn, including root parts, remained intact. We also carefully evaluated all areas of the synovium and noted that no acute synovitis was present. Little fibrotic tissue was noted at Hoffa’s tissue and was excised. After the diagnostic procedure, an additional anteromedial portal (far medial portal) was created. The deficient site of the meniscus body was excised to the healthy region, including the peripheral rim. The tear size was then measured. Two tibial bone tunnels were created at the posterior and anterior borders of the deficient site. The tendon graft was cut to 110% of the measured length. The posterior and anterior parts were prepared using two simple sutures (fiberwire). The other limb of the simple suture was then passed through the native tissue. The tendon graft was drifted into the joint. After positioning the graft, multiple all-inside sutures were performed. A suture anchor for centralization was inserted to prevent graft extrusion. The suture limbs were carried through the bone tunnel and secured with a button on the tibial side (Figure 3a–c and Figure 4).

After completing the meniscus transplantation, the scope was shifted into the anteromedial (AM) portal. The outside-in ACL drill guide (Smith & Nephew) was introduced from the AL portal with the tip placed at the site of the anteromedial bundle (AMB) femoral footprint. Outside the joint, the accompanying drill sleeve was positioned just proximally and posteriorly to the lateral femoral epicondyle. Following guide pin placement, an 8 mm femoral tunnel was created using a rigid reamer in an outside-in manner.

The remaining peroneal longus tendon graft was divided into two parts; one was triple-folded, and the other part was used for anterolateral ligament reconstruction (non-folded). Both grafts were passed through the tibial and femoral bone tunnels together. The non-folded graft was shuttled through the tibial ACL tunnel and pulled out from the femoral tunnel until an ideal length was sufficient for creating an extraarticular anterolateral ligament (ALL). After drilling the tibial ALL tunnel, the graft was passed under the iliotibial band and through the tibial ALL tunnel. Both the femoral ACL and tibial ALL tunnels were secured with interference screws (Figure 3d).

## 4. Rehabilitation

Partial weight-bearing was allowed for the first six weeks with crutches assistance. A hinged knee brace was used, set at 0–30° for two weeks, then gradually increased by 30° every two weeks. Unrestricted range of motion (ROM) was permitted two months after surgery.

We had recorded the International Knee Documentation Committee score (IKDC), the Marx activity score, and Single Assessment Numeric Evaluation (SANE) preoperatively and 1 year postoperatively. The patient reported outcome measure (PROMS) data is listed in Table 2.

## 5. Discussion

Meniscus allograft transplantation has shown good mid- to long-term outcomes, making it rare to use tendinous autografts for segmental meniscus loss in previous studies [16]. This case report aimed to describe a case of recurrent traumatic MM tear with ACL rupture, which was treated using a segmental meniscal transplantation technique. The surgery involved a single peroneus longus tendon autograft with simultaneous ACL reconstruction, representing a novel approach to meniscal transplantation surgery.

Due to the high cost, the need for size matching, and various legal regulations across countries, autologous tendon grafts have been explored as an alternative to costly allograft meniscus transplantation since 2000. However, a preliminary study involving five patients (two males and three females with an average age of 41 years) who underwent autologous tendon meniscus transplantation for lateral meniscus defects and severe degenerative arthritis of the knee joint showed that the tendon grafts did not successfully replace the missing meniscus cartilage. Only one patient experienced minimal clinical improvement, while the others did not improve [17].

As the initial results were poor and allograft meniscus transplantation has been successful, there has not been much progress in autologous tendon meniscus transplantation. Nonetheless, animal experiments have continued to explore this approach, such as the study published by Nobutake Ozeki in *Stem Cells* in 2015 [18], which investigated the ability of mesenchymal stem cell (MSC) injection into the tendon grafts to prevent cartilage degeneration in a rat with a partial meniscus defect model. The study showed that regardless of MSC use, the tendon grafts increased meniscus size, and MSCs promoted meniscus regeneration and prevented cartilage degeneration in the rat model.

Another study published by Jianning Zhao et al. [19] in 2017 implanted autologous tendon grafts treated with kartogenin (KGN) or saline into the rabbit knee after removing part of the meniscus. Histological analysis and immunostaining were performed to examine the formation of the meniscus. After three months of implantation, the tendon grafts treated with KGN showed a structure similar to the normal meniscus and contained many cartilage-like cells and fibrocartilage-like tissue.

In 2022, Hiroaki Nakamura published a study investigating the use of bone marrow aspirate (BMA) injection to enhance the histological properties of autologous tendon as a meniscus graft [20]. And, they concluded that the autogenous tendon grafts by injecting BMA improved the histologic score of the regenerated meniscal tissue. 

Recently, Karl Eriksson and colleagues published their results on using semitendinosus tendon in a total meniscal transplant [21]. Seven patients were included in the study, with six undergoing medial meniscal transplantations and one undergoing lateral meniscal transplantation, with a mean age of 29 years. Four patients were followed up with for 12 months. The results showed improvements in International Knee Documentation Committee score (IKDC), Knee injury and Osteoarthritis Outcome Score (KOOS), and Lysholm scores. MRI demonstrated that the transplants became more wedge-shaped, and meniscal root formation was observed. The authors believe that tendon grafts for meniscal transplant can survive and produce the shape and function of the original meniscus, leading to improvements in pain and quality of life for patients.

Kirill Baranov, M.D., recently proposed another technique using autologous peroneal longus as a meniscal transplant, and MRI also showed significant wedge-shaped tissue formation [14]. This report highlights a technique for lateral meniscus deficits using autografts derived from the peroneus longus tendon. The peroneus longus tendon autograft demonstrates promising safety and efficacy, making it a viable option for preventing osteoarthritis of the lateral knee aspect. Notably, size measurement is less critical, as excess tissue can be easily managed. 

According to the abovementioned studies, using autologous tendon graft for partial or total meniscus transplant may become an alternative choice in the future, as surgical techniques and orthobiologic treatments gradually improve. Further research, including clinical and biomechanical studies, is necessary to evaluate the effectiveness of segmental meniscus reconstruction using autologous tendon grafts. Though this patient did not have issue of synovitis, it was still necessary to conduct a larger study to evaluate the impact of synovitis and postoperative inflammation on the tendon graft. Continued investigation in this area could potentially lead to improved and more accessible treatment options for patients with segmental meniscal loss, addressing the current challenges associated with cost and size matching.

## 6. Conclusions

While total meniscus transplant using autologous tendon graft has become more popular, there are limited studies discussing the use of autograft for segmental meniscal loss. Typically, segmental meniscus deficits are treated with either meniscus scaffold or meniscus allograft [22,23]. However, segmental reconstruction using either allograft or scaffold still faces challenges related to cost and size matching.

We present a case of a patient who experienced a re-rupture of the anterior cruciate ligament (ACL) following previous ACL reconstruction, as well as multiple failed attempts of meniscal repair. In this case, we utilized the autogenous peroneal longus tendon as a graft for ACL combined anterolateral ligament reconstruction, combined with segmental meniscal transplant. The patient achieved excellent clinical outcomes. These findings highlight the potential efficacy of using tendon grafts for meniscal transplant, and we believe further clinical and biological research is warranted to validate this approach.

## Figures and Tables

**Table 1:** Demographic data.

Age	26
Sex	Male
Height	188 cm
Weight	97 kg
BMI	27.44
Race	Asian
Occupation	Student
Physical examination during admission	
Range Of motion	Right 10–120 degrees Left 0–135 degrees
Anterior drawer test	Right Positive, grade 2 Left Negative
Pivot shift test	Right Positive, grade 3 Left Negative
McMurray test	Right Positive Left Negative

**Table 2:** PROMS data of this case.

	Preoperative	Postoperative (1 Year)
IKDC	44.8	93.1
Marx activity score	0	10
SANE score	45	88

## Data Availability

The dataset supporting the conclusions of this article is available from the corresponding author on reasonable request.

## References

[1] Ozeki N., Seil R., Krych A.J., Koga H. (2021). Surgical treatment of complex meniscus tear and disease: State of the art. J. ISAKOS Jt. Disord. Orthop. Sports Med..

[2] Fox A.J., Wanivenhaus F., Burge A.J., Warren R.F., Rodeo S.A. (2015). The human meniscus: A review of anatomy, function, injury, and advances in treatment. Clin. Anat..

[3] Collins J.E., Shrestha S., Losina E., Marx R.G., Guermazi A., Jarraya M., Jones M.H., Levy B.A., Mandl L.A., Williams E.E. (2022). Five-Year Structural Changes in the Knee Among Patients With Meniscal Tear and Osteoarthritis: Data From a Randomized Controlled Trial of Arthroscopic Partial Meniscectomy Versus Physical Therapy. Arthritis Rheumatol..

[4] Persson F., Turkiewicz A., Bergkvist D., Neuman P., Englund M. (2018). The risk of symptomatic knee osteoarthritis after arthroscopic meniscus repair vs partial meniscectomy vs the general population. Osteoarthr. Cartil..

[5] Olivotto E., Belluzzi E., Pozzuoli A., Cigolotti A., Scioni M., Goldring S.R., Goldring M.B., Ruggieri P., Ramonda R., Grigolo B. (2022). Do Synovial Inflammation and Meniscal Degeneration Impact Clinical Outcomes of Patients Undergoing Arthroscopic Partial Meniscectomy? A Histological Study. Int. J. Mol. Sci..

[6] Olivotto E., Trisolino G., Belluzzi E., Lazzaro A., Strazzari A., Pozzuoli A., Cigolotti A., Ruggieri P., Evangelista A., Ometto F. (2022). Macroscopic Synovial Inflammation Correlates with Symptoms and Cartilage Lesions in Patients Undergoing Arthroscopic Partial Meniscectomy: A Clinical Study. J. Clin. Med..

[7] Saltzman B.M., Cotter E.J., Wang K.C., Rice R., Manning B.T., Yanke A.B., Forsythe B., Verma N.N., Cole B.J. (2020). Arthroscopically Repaired Bucket-Handle Meniscus Tears: Patient Demographics, Postoperative Outcomes, and a Comparison of Success and Failure Cases. Cartilage.

[8] Lyman S., Hidaka C., Valdez A.S., Hetsroni I., Pan T.J., Do H., Dunn W.R., Marx R.G. (2013). Risk factors for meniscectomy after meniscal repair. Am. J. Sports Med..

[9] Frank R.M., Cole B.J. (2015). Meniscus transplantation. Curr. Rev. Musculoskelet Med..

[10] Heckmann T.P., Barber-Westin S.D., Noyes F.R. (2006). Meniscal repair and transplantation: Indications, techniques, rehabilitation, and clinical outcome. J. Orthop. Sports Phys. Ther..

[11] Yanke A.B., Huddleston H.P., Chahla J., Cole B.J. (2022). Medial Meniscus Transplantation and Bone-Tendon-Bone Anterior Cruciate Ligament Reconstruction. J. Am. Acad. Orthop. Surg..

[12] Seiter M.N., Haber D.B., Ruzbarsky J.J., Arner J.W., Peebles A.M., Provencher M.T. (2021). Segmental Meniscus Allograft Transplantation. Arthrosc. Tech..

[13] Ramme A.J., Strauss E.J., Jazrawi L., Gold H.T. (2016). Cost effectiveness of meniscal allograft for torn discoid lateral meniscus in young women. Phys. Sportsmed..

[14] Milenin O., Strafun S., Sergienko R., Baranov K. (2020). Lateral Meniscus Replacement Using Peroneus Longus Tendon Autograft. Arthrosc. Tech..

[15] Nordin J.S., Olsson O., Lunsjö K. (2019). The gracilis tendon autograft is a safe choice for orthopedic reconstructive procedures: A consecutive case series studying the effects of tendon harvesting. BMC Musculoskelet Disord..

[16] Zaffagnini S., Grassi A., Marcheggiani Muccioli G.M., Benzi A., Roberti di Sarsina T., Signorelli C., Raggi F., Marcacci M. (2016). Is Sport Activity Possible After Arthroscopic Meniscal Allograft Transplantation?: Midterm Results in Active Patients. Am. J. Sports Med..

[17] Johnson L.L., Feagin J.A. (2000). Autogenous tendon graft substitution for absent knee joint meniscus: A pilot study. Arthroscopy.

[18] Ozeki N., Muneta T., Matsuta S., Koga H., Nakagawa Y., Mizuno M., Tsuji K., Mabuchi Y., Akazawa C., Kobayashi E. (2015). Synovial mesenchymal stem cells promote meniscus regeneration augmented by an autologous Achilles tendon graft in a rat partial meniscus defect model. Stem Cells.

[19] Huang H., Xu H., Zhao J. (2017). A Novel Approach for Meniscal Regeneration Using Kartogenin-Treated Autologous Tendon Graft. Am. J. Sports Med..

[20] Iida K., Hashimoto Y., Orita K., Nishino K., Kinoshita T., Nakamura H. (2022). The Potential of Using an Autogenous Tendon Graft by Injecting Bone Marrow Aspirate in a Rabbit Meniscectomy Model. Int. J. Mol. Sci..

[21] Rönnblad E., Rotzius P., Eriksson K. (2022). Autologous semitendinosus tendon graft could function as a meniscal transplant. Knee Surg. Sports Traumatol. Arthrosc..

[22] Haber D.B., Douglass B.W., Arner J.W., Miles J.W., Peebles L.A., Dornan G.J., Vidal A.F., Provencher C.M.T. (2021). Biomechanical Analysis of Segmental Medial Meniscal Transplantation in a Human Cadaveric Model. Am. J. Sports Med..

[23] Houck D.A., Kraeutler M.J., Belk J.W., McCarty E.C., Bravman J.T. (2018). Similar clinical outcomes following collagen or polyurethane meniscal scaffold implantation: A systematic review. Knee Surg. Sports Traumatol. Arthrosc..

